# Treatment of inborn errors of metabolism

**DOI:** 10.1186/1755-8166-7-S1-I42

**Published:** 2014-01-21

**Authors:** Anil B Jalan

**Affiliations:** 1 Navi Mumbai Institute of Research in Mental and Neurological Handicap, C-116, Om Rachna Society, Sector 17, Vashi, Navi Mumbai, India

## 

Inborn errors of metabolism (IEM), though individually rare are collectively common. Average incidence of 50+ common IEMs is considered to be approx 1 in 1,000 live births. With annual birth rate of approximately 25 million babies in India, we can expect at least 25,000 babies being born with IEM in India and hence it is a significant burden to the families and societies. Over the last 3 decades many new and effective therapies have emerged for the management and treatment of IEMs. As of today approximately 70 different forms of therapeutic agents are available to help people suffering from IEM. Besides these we have various forms of transplants–liver cell transplants, liver transplant (both ALT and OLT), bone marrow or HSCT and kidney transplant now easily available at various parts of India. Amongst hundreds of IEM, certain disorders are common and treatable with simple forms of therapeutic agents. Urea Cycle Disorders and Organic Acidemias are top on the list. Every pediatrician and neonatologist must be aware of emergency management of these two group of disorders as they may present at any age especially in the 1^st^ decade and more so in infancy. Both of these groups of disorders may present with hyperammonemia as their first manifestations and needs to be treated with easily available medications as oral form of Sodium Benzoate (250–500 mg/kg/day in 2-3 divided doses), low protein diet or temporary stoppage of protein intake till acute crisis is under control. For UCDs one can use arginine (granules or powder) hydrochloride or base–250 mg/kg/day in 2-3 divided doses except for Arginase deficiency. For disorders like CPS and NAGS deficiency Carbaglu of Carbamylglutamate or N Acetyl Glutamate can also be used in the dose of 100–300 mg/kg/day. Now a days it is also recommended for treatment of certain organic acidemias e.g. propionic acidemia. Other products like Sodium Phenyl butyrate or injectable forms of Arginine (10%) or Sodium Benzoate+Sodium Phenylbutyrate) are not available in India and are very costly for an average Indian patient. Once acute crisis is managed, special diets with low protein are successful in managing most of the UCDs. For Organic acidemias like Propionic acidemia, Methyl malonic Acidemia and Isovaleric acidemia, L-Carnitine in the dose of 100–300 mg/kg/day must be used. Injectable form of L-Carnitine is also available for emergency management. Correction of acidosis is very important along with supplementation of adequate amount of dextrose. One can use Dextrose-Insulin drip in emergency. Besides these, other medications like Betaine, NTBC, Dextromethrophane, Diazoxide, certain vitamins e.g. Biotin, Vit-B12, Thiamine, Riboflavin, Folic acid, Folinic acid, Pyridoxine, Pyridoxal–5-Phosphate, Vit C, certain aminoacids like Glycine, Ornithine, Citrulline etc are also available for the management of various types of IEMs. Of late many enzymes are available for enzyme replacement therapies of LSDs e.g. Gaucher, Pompe, Fabry’s Disease, MPS I, II and VI.

